# Progress in Surgery

**Published:** 1897-01-09

**Authors:** 


### PROGRESS IN SURGERY.

### DISEASES OF THE UPPER AIR PASSAGES.

(*Continued from page 234.*)

**Diseases of the Nose and Throat in Relation to Disorders of Digestion.**—T. R. French[4] states that the causes of chronic catarrhal pharyngitis mentioned in text-books on diseases of the throat, nose, and ear, and in monographs on pharyngitis, are very numerous. The conditions and diseases which are given the most prominence, and upon which accepted authorities are more or less agreed, are: Frequent attacks of acute pharyngitis, extension of catarrhal inflammation from the nasal passages or naso-pharynx, nasal stenosis, nasal polypi, the excessive use of tobacco or alcohol, improper use of the voice, reflected irritation from a disordered digestion or diseases of the genito-urinary tract, diseases of the glandular and nervous systems, congested portal venous circulation, lithæmia, rheumatic and gouty diathesis, dust, smoke, in which acid fumes are inhaled, and sudden changes of hot and cold foods. While it is possible that all the above-mentioned conditions may play important parts as factors in the production or aggravation of catarrhal pharyngitis in certain cases, his belief is that in a very large majority of cases the cause of this disease will be found in some portion of the gastro-intestinal tract. Except in a few of those who have grossly abused the vocal apparatus in speaking and singing, he does not recall having examined a patient with chronic catarrhal or follicular pharyngitis in whom some disturbance of the digestion could not be detected. One rarely, if ever, exists without the other, and this is in itself highly suggestive of a distinct relationship, and, perhaps, interdependence. It seems to French reasonable to suppose that many of the diseases and conditions mentioned as causes of pharyngeal catarrh may act indirectly as factors by first causing the disorder of the digestion. Naso-pharyngeal catarrh is commonly attributed to the climate and its changeable character, to unclean and dusty cities, and to various other conditions. In comparison with the inhabitants of other countries, there is probably no country in which disturbances of the digestive function are more common than in America. Catarrhal affections of the upper air-passages are known to be most prevalent where the climatic changes are the greatest; but French believes it will be found that catarrh of the pharynx, at least, prevails in communities where the general air of activity causes the habit of eating in haste and at irregular intervals. From a clinical standpoint there is reason for the belief that functional disorders of the digestive tract are capable of producing vasomotor reflex irritation of the inferior turbinated bodies, as is evidenced by swelling of those bodies at certain times, as, for instance, on rising in the morning, and during attacks of acute gastric or duodenal catarrh. The proof of the connection is not only in the occurrence of the two affections at the same time, but also in the fact that the nasal irritation disappears after the acute indigestion has passed, or is diminished after improvement of the chronic condition of the stomach. The author cites Beverley Robinson's paper on "Dyspepsia as reflected in the mucous membrane of the upper air-passages," which was read before the American Laryngological Association in 1888, in which he suggests the probability of the muco-pus from a catarrh of the upper air-passages being capable of exciting a gastric disturbance. Such a relationship of cause and effect seems to French to be highly plausible. It is a mooted question as to whether the discharge from a chronic catarrhal inflammation in the nose is irritating in its character.

It is that class of voice-users, where there is to be found an underlying catarrhal condition of the pharynx and larynx of mild degree, which we, as physicians, are most called upon to treat for impairment of the vocal function. If, as French believes, the underlying cause of the catarrh is in the majority of cases an impaired digestion, we render our patients but poor service unless we extend the treatment to parts lying far below the throat.

The same subject has been discussed by M. R. Brown,[5] of Chicago. He called attention to the pharyngeal hyperæmia so often found in cases of stomach cough. Asthmatic attacks were frequently due to digestive disorders, which also caused œdema of the larynx, which might be angio-neurotic in character. Allusion was made also to the various acute throat conditions seen in the course of hepatic cirrhosis. On the other hand, gastric disturbance often came from swallowing the secretions from sores in the pharynx. Glottic spasm might result from upward pressure of the diaphragm caused by gastro-enteric troubles. Much attention had recently been given to the laryngeal condition in typhoid fever. We might have all grades of severity of lesions, from simple hyperæmia up to loss of tissue, œdema, infiltration, ulceration, or perichondritis. There was no proof of the direct influence of the stomach in many throat conditions, but there was a strong clinical suggestion of the relation of cause and effect.

In connection with these recent articles we may cite Watson Williams'[6] remarks on dyspepsia as a cause of chronic pharyngitis. He states that dyspepsia, especially if associated with constipation and portal congestion, is a very common cause. Chronic portal congestion, whether due to gastro-intestinal catarrh or heart disease, results in a chronic congestion of the pharyngeal mucous membrane. But there are many cases, he continues, that must be regarded as toxic, and due to a sluggish liver failing to arrest and destroy the toxic products of imperfect digestion, which have a specific effect on the pharynx and fauces like muscarin and belladonna. The redness and injection, the dryness of the throat and pain in deglutition, and sense of roughness or presence of a foreign body in the pharynx, which are characteristic of belladonna or muscarin poisoning, are exactly imitated in dyspeptic pharyngitis, and though these symptoms often occur in a mild degree, their frequent repetition accounts for the permanent alterations in the structure of the mucous membrane. He accounts for the pharyngitis due to lithiasis in a similar manner.

[4] Commun. to the Amer. Laryng. Assoc.; New York Med. Journ., Sept. 12, 1896. [5] Trans. of the Amer. Laryng. Assoc., Pittsburg, May, 1896; Journ. of Laryng., Aug., 1896. [6] Diseases of the Upper Respiratory Tract, American edition, 1894, p. 32, *et seq.*

### SURGERY OF THE VERMIFORM APPENDIX.

Klecki[1] has shown—by strangulating a portion of intestine in the dog—that a virulent multiplication of the organisms which are normally found in the intestine takes place, and that peritonitis occurs under twenty-four hours without any perforation of the occluded gut. From this Dieulafoy[2] concludes that the immediate predisposing cause of an attack of appendicitis is the closure of the appendicular canal. This may be brought about by the slow and progressive growth of a calculus; under the influence of a non-calculous inflammation by the swelling of the mucous membrane; by calculus and swelling conjoined; or by a slow formation of a fibrous stricture in some portion of the appendix. The canal may be closed either at its cæcal extremity or in some part of its course. Following on this closure, we have a multiplication and increased virulence of the contained organisms as in the experiment of Klecki referred to above. Poucet,[3] having studied fifteen appendices removed by operation, says, however, that this theory of Dieulafoy does not hold good in the majority of cases, and that temporary or permenent obstruction of the vermiform appendix cannot be regarded as a necessary condition of appendicular colic and abscess. The contraction in an inflamed appendix is the result, and not the cause, of the disease. The only cause of appendicitis, which has been proved with certainty, is infection, which gives to the disease its peculiar character according to the virulence of the infection, The author also thinks with regard to heredity and family predisposition to appendicitis, that it is more a question of soil. The hereditary theory is supported by Dieulafoy,[4] who appears[5] to be convinced of the necessity of speedy operative interference in all cases of confirmed appendicitis. Pozzi,[6] too, thinks that whenever an appendicitis causes local or general symptoms, no matter what degree of severity, the appendix should at once be removed, because these symptoms may immediately endanger the life of the patient, or if temporarily relieved, may leave him the victim of a painful infirmity, which constitutes a threatening danger. Speaking of the treatment of the milder forms of appendicitis, Lane[7] says morphia must be given in sufficient quantity to keep the pain in check. If there is matting of the intestines about the appendix there is rarely much cause for anxiety, as perforation is unusual in these cases, and abscesses generally spread in the direction of the abdominal wall. Unless symptoms are urgent, there is no hurry to open these fluid accumulations, but when opened they must be treated with strict antiseptic precautions in spite of any fæcal odour, for the bacillus coli communis does not seem to impede healing if the septic germs are kept from the wound. The author evacuates as much pus as he can, and then packs freely with sterile iodoform gauze, having previously passed sutures through the several muscular planes of the abdominal wall. This is done because of the difficulty there may be later of recognising the different layers. Within twenty-four hours the edges of the wound are brought accurately together.

In the discussion on appendicitis at the recent meeting of the British Medical Association, MacDougall[8] thought that the cases might be grouped into four classes—(1) Mild cases; (2) cases going on to the formation of abscess; (3) cases resulting in perforation; and (4) relapsing cases. Surgical treatment is not needed in many cases. In the perforative variety the only chance is in operation; and, therefore, a quick, thready pulse, marked abdominal distension, acute pain, and extreme tenderness are signs in favour of immediate interference. The rule, in children, should be to operate early. Before the first forty-eight hours operation is easier than later on, but each case must be decided on its merits. When the abdomen has to be opened, and pus is present without general peritonitis, the peritoneal cavity can be easily shut off by means of sponges. It is not good surgery to search for and remove the appendix in every case; if the appendix presents itself it may be removed, but if it be not obvious, or if it form part of the wall of an abscess, it should be left. If intestinal obstruction has occurred before the opening of an abscess, the abscess cavity should be dealt with before the adhesions which are compressing the bowel are broken down. In recurrent appendicitis the appendix should be removed, but it is not always possible to do so. In the removal of an appendix a peritoneal flap should be formed, as simple ligature is unsafe. Rutherford Morison[9] would operate in the following cases: (1) After a second attack; (2) cases accompanied by abscess; (3) all cases of perforation; (4) the sudden onset of urgent symptoms in quiescent cases. In abscess around the appendix on the inner aspect of the cæcum, drainage only, without removal of the appendix, is the best treatment. The incision in the linea semilunaris is bad as the nerves to the rectus are cut, causing some paralysis of muscle predisposing to hernia, and drainage is difficult. Morton[10] and Jordan Lloyd[11] speak in favour of early operation, and the latter considers that the cases can best be classified according to the position of the free end of the appendix. 1. If the appendix is in the iliac fossa the classical symptoms will be present, and in this form, but no other, there is an iliac swelling. 2. If the tip of the appendix drop into the pelvis there will be no iliac swelling, but a swelling can be felt per rectum. 3. In the lumbar variety the appendix has swung back into the loin, where a fulness can be felt. 4. In the last variety the appendix projects into the abdominal cavity, and the swelling is abdominal; and resonance can often be obtained on percussion between the swelling and the groin. In the pelvic variety an abscess is best drained through the vagina in the female and through the rectum in the male. Renton[12] and Bush[13] believe it to be unwise to search for the appendix in all cases. Mitchell Banks[14] says that inasmuch as the great bulk of pericæcal or appendical abscesses tend to push forwards and outwards, the incision should not be vertical but transverse, and should reach well back. When the muscles have been divided the surgeon should bore with his finger in the direction in which he thinks the pus is. He may come upon it quite behind the peritoneum, and then a large glass drainage tube is all that is required. If the pus is intra-peritoneal, then the peritoneum can be easily opened by prolonging the incision towards the median line. The bowels can be readily kept back with sponges or gauze, and the appendix removed if diseased, while any pent-up pus can be let loos[illegible]

washed out. Thorough flushing with hot water is most important. White[15] thinks an operation is indicated whenever the onset of a case of appendicitis is sudden and severe; whenever, during even a mild attack, the symptoms have not lessened in severity at the end of forty-eight hours; whenever, in cases seen later, a firm, slowly-forming, well-defined mass can be felt in the right iliac fossa; whenever a sudden increase in the acuteness of the pain and a rapid diffusion of tenderness occurs; whenever the disease is supposed to be of tuberculous origin; whenever attacks have been numerous or increasing in gravity, or have unfitted the patient for work, or have caused persistent local symptoms, or have at any time put the patient's life in great danger. When there is a circumscribed abscess, the author thinks it is poor surgery to insist on removing the appendix in the face of all obstacles. Of thirty-seven cases in which he had operated and left the appendix there was not a single fatal result. A fæcal fistula formed in two cases, but closed spontaneously in both. In three cases there was a recurrence; but in two of these the appendix was removed with ease, and in the third it had become obliterated. In his views as regards the necessity and desirability of leaving the appendix in some cases, the author is supported by McBurney,[16] Senn,[17] Halsted,[18] Richardson,[19] and Bull.[20] McBurney[21] says one may meet with two classes of abscess which differ in the necessary management. In one kind, the abscess approaches the anterior abdominal wall, and the abscess is opened without opening the general peritoneal cavity. In such a case, where the appendix forms an integral part of the abscess wall, any attempt at removal of the appendix would be liable to produce a general infection of the peritoneum, and must not, therefore, be undertaken. In the other variety, the abscess has not approached the anterior abdominal wall, the pus and appendix being surrounded by coils of matted intestine, and the whole mass lying quite free in the general abdominal cavity. In operating on such a case, one deliberately opens the general peritoneal cavity, and then the whole area involved can be isolated with gauze or sponge packing, so as to exclude the non-infected portion of peritoneum from the field of operation during the entire procedure. The coils of intestine which enclose the appendix can then be unravelled, and the appendix removed. Cleveland[22] says the best method of treatment in the early stage of appendicitis is by the administration of Epsom salts, but the treatment must be adopted within the first twenty-four or forty-eight hours. Johnson[23] advocates the removal of the appendix in all operative cases; but Parker[24] adopts the more conservative attitude referred to above. McBurney[25] records a successful operation for volvulus ten days after operation for appendicitis, and also a case of secondary operation for extensive adhesions following appendicitis. Other interesting cases of operation for appendicitis are reported by Sheen,[26] Moynihan,[27] Morison,[28] Smith,[29] Nicaise,[30] and Elder.[31]

[1] "Recherches sur la Pathogénie de la Peritonite d'Origine Intestinale: étude sur la Virulence de Coli-bacille." Ann. de l'Instit. Pasteur, Vol. IX., pp. 710-736. [2] Clin. Journ., Sept. 9th, 1896, p. 310, *et seq.* [3] Med. Week, May 22nd, 1896. [4] Loc. cit. [5] Med. Week, May 1st, 1896. [6] Ibidem. [7] Lancet, July 25th, 1896, p. 228. [8] Med. Rec., New York, Aug. 29th, 1896, p. 311; Lancet, Aug. 9th, 1896, p. 423; and Brit. Med. Journ., Oct. 10th, 1896, p. 997, *et seq.* [9] Ibidem. [10] Ibidem. [11] Ibidem. [12] Ibidem. [13] Ibidem. [14] Brit. Med. Journ., Oct. 10th, 1896, p. 1,007. [15] Amer. Journ. Med. Sci., June, 1896, and Med. Mag., March, 1896. [16] Ibidem, and Annals of Surg., June, 1896. [17] Ibidem. [18] Ibidem. [19] Ibidem. [20] Ibidem. [21] Annals of Surg., June, 1896, p. 750. [22] Chicago Clinic. Rev., Aug., 1896. [23] Med. Rec., New York, May 23rd, 1896. [24] Ibidem. [25] New York Med. Rec., July 25th, 1896. [26] Practitioner, June, 1896. [27] Brit. Med. Journ., June 6th, 1896. [28] Med. Press and Circul., June 3rd, 1396. [29] Edin. Med. Journ., Aug., 1896. [30] Glasgow Med. Journ., Aug., 1896, and Rev. de Chir., May, 1896. [31] Lancet, Oct. 10th, 1896.

